# Analysis of risk factors for hepatic sinusoidal obstruction syndrome following allogeneic hematopoietic stem cell transplantation in pediatric patients

**DOI:** 10.1007/s00432-021-03732-1

**Published:** 2021-07-13

**Authors:** Jaspar Kloehn, Grit Brodt, Jana Ernst, Bernd Gruhn

**Affiliations:** Department of Pediatrics, Jena University Hospital, Am Klinikum 1, 07747 Jena, Germany

**Keywords:** Allogeneic hematopoietic stem cell transplantation, Sinusoidal obstruction syndrome, Children, Adolescents and young adults, Risk factors, International normalized ratio

## Abstract

**Purpose:**

Hepatic sinusoidal obstruction syndrome (SOS) represents a serious complication following hematopoietic stem cell transplantation (HSCT). Our study aimed to investigate important risk factors of SOS in a pediatric population.

**Methods:**

This retrospective study analyzed 105 children, adolescents and young adults who underwent allogeneic HSCT at our pediatric HSCT center in Jena. The observation period was 12 years and SOS was defined by the pediatric criteria of the European Society for Blood and Marrow Transplantation (EBMT).

**Results:**

15 out of all 105 patients developed SOS (14.3%). The median time from HSCT to SOS diagnosis was 12 days. The mortality rate of SOS was 20.0%. In univariate analyses, we identified the significant risk factors of patient age < 1 year [odds ratio (OR) = 7.25, *p* = 0.037], prior treatment with gemtuzumab ozogamicin (OR = 11.00, *p* = 0.020), high pretransplant ferritin levels above 1500 ng/mL (OR = 4.00, *p* = 0.033), 2000 ng/mL (OR = 4.69, *p* = 0.016), and 2400 ng/mL (OR = 5.29, *p* = 0.005) as well as international normalized ratio (INR) ≥ 1.3 (OR = 5.91, *p* = 0.009). The following risk factors could be confirmed in multivariate analysis: treatment with gemtuzumab ozogamicin (OR = 9.24, *p* = 0.048), ferritin > 2400 ng/mL (OR = 5.74, *p* = 0.023), and INR ≥ 1.3 (OR = 8.02, *p* = 0.007).

**Conclusion:**

Our study confirms several risk factors from the current literature. Additionally, this is the first report on the risk factor of high pretransplant INR.

## Introduction

Hepatic sinusoidal obstruction syndrome (SOS), previously called veno-occlusive disease (VOD), is among the potentially life-threatening complications following hematopoietic stem cell transplantation (HSCT).

Pathophysiologically, initial damage to the sinusoidal endothelium leads to an activation of endothelial cells (DeLeve et al. 1999, 2002). This damage is caused by factors like chemotherapy or radiotherapy as part of the conditioning regimen before HSCT. The unregulated endothelial activation results in a loss of sinusoidal barrier, leading to extravasation of erythrocytes, leukocytes, and cellular debris into the space of Disse. Moreover, a cascade of thrombotic and antithrombotic effects causes a hemostatic imbalance. The damaged sinusoids induce a downstream embolization, sinusoidal obstruction, and occlusion of terminal hepatic venules (Carreras and Diaz-Ricart 2011; Coppell et al. 2003; Mohty et al. 2015).

The frequency of SOS varies widely in the published literature depending on different diagnostic criteria (Carreras et al. 2011; Coppell et al. 2010; Kammersgaard et al. 2019). Coppell et al. (2010) showed a mean incidence of 13.7% for SOS following HSCT by analyzing different reports of SOS occurring with a range from 0 to 62%. Traditional diagnostic standards are based on the Baltimore criteria, reported by Jones et al. (1987), or the Seattle criteria, reported by McDonald et al. (1984). Recently, new diagnostic criteria have been published on behalf of the European Society for Blood and Marrow Transplantation (EBMT) to achieve an earlier identification and to detect late-onset SOS. Mohty et al. (2016) developed the EBMT criteria for SOS in adult patients. Furthermore, Corbacioglu et al. (2018) published the pediatric EBMT criteria. These criteria depend on the clinical findings transfusion-refractory thrombocytopenia, unexplained weight gain on three consecutive days or weight gain > 5%, hepatomegaly, ascites, and hyperbilirubinemia (≥ 2 mg/dL). In addition to the EBMT criteria, Cairo et al. (2020) proposed modified diagnostic criteria. In the past years, different criteria for severity grading were published (Cairo et al. 2020; Corbacioglu et al. 2018; McDonald et al. 1993; Mohty et al. 2016). Common to all, the most severe form of SOS can lead to multi-organ dysfunction with a mortality rate of up to 84% (Coppell et al. 2010).

The most promising therapeutic option for SOS is the use of defibrotide, which was shown in several studies for both adult and pediatric patients (Corbacioglu et al. 2016; Richardson et al. 2016). Additionally, the prophylactic effect of defibrotide was described (Corbacioglu et al. 2012; Qureshi et al. 2008).

The known risk factors for SOS can be classified into patient-related factors and transplantation-related factors (Corbacioglu et al. 2019; Dalle and Giralt 2016). Former factors include young patient age, preexisting liver disease, advanced malignant underlying diseases, treatment with gemtuzumab ozogamicin, high transaminase levels, high serum ferritin, and genetic factors (Carreras et al. 1998; Cheuk et al. 2007; Maximova et al. 2014; Morado et al. 1999; Seifert et al. 2015; Wadleigh et al. 2003). Reported transplantation-related risk factors are allogeneic HSCT, conditioning regimen based on busulfan, cyclophosphamide, fludarabine or total body irradiation, and unrelated donors (Barker et al. 2003; Carreras et al. 1998; Carreras et al. 2011).

Even though some risk factors are already known, it is important to confirm these results and to analyze new potential risk factors. This will lead to better risk stratification and earlier identification of SOS. The purpose of our study was to evaluate the risk factors of SOS in pediatric patients undergoing allogeneic HSCT.

## Patients and methods

### Patients

Our retrospective study included 105 children, adolescents and young adults (AYA) who underwent allogeneic HSCT at the Department of Pediatrics of Jena University Hospital in Jena, Germany. We only analyzed recipients with the first HSCT. Patients who received defibrotide prophylaxis were excluded. The transplantations were performed between January 2007 and December 2018. All patients underwent a myeloablative conditioning regimen and were nursed in single rooms with a laminar airflow filtration system.

### Definitions

SOS was defined by using the pediatric EBMT criteria (Corbacioglu et al. 2018). These criteria include transfusion-refractory thrombocytopenia, unexplained weight gain on 3 consecutive days or weight gain > 5%, hepatomegaly, ascites as well as bilirubin ≥ 2 mg/dL or rising bilirubin on 3three consecutive days. The diagnosis of SOS was confirmed when at least 2 of the mentioned criteria were met without any limitation for the time of onset. The classification of the severity of SOS was based on the pediatric severity criteria of the EBMT and consisted of mild, moderate, severe, and very severe SOS (Corbacioglu et al. 2018). Following criteria were included

: rise of liver biomarkers, persistent refractory thrombocytopenia, rise and kinetics of bilirubin, ascites, dysfunctional coagulation as well as renal, pulmonary, or cognitive impairment.

### Risk factors

In our study, we considered patient-related factors including several laboratory parameters and transplantation-related factors. Some analyzed factors were already known to be associated with SOS. Moreover, we investigated new potential risk factors. The following transplantation-related factors were included in our study: conditioning regimen based on busulfan, cyclophosphamide, melphalan or total body irradiation, graft source, donor age, donor sex, and donor-recipient human leukocyte antigen (HLA)-match. In addition, we explored the following patient-related factors: patient age, patient sex, prior treatment with gemtuzumab ozogamicin as well as the laboratory parameters of aspartate transaminase, alanine transaminase, cholinesterase, glutamyl transpeptidase, lactate dehydrogenase, alkaline phosphatase, ferritin, albumin, total bilirubin, C-reactive protein and international normalized ratio (INR). All laboratory values were determined before HSCT. Furthermore, cutoffs were chosen for metric variables such as age and laboratory parameters. These cutoffs were defined by reference values, clinical consideration, and receiver operating characteristic (ROC) curve analysis.

### Statistical analysis

To evaluate the association between the analyzed factors and the occurrence of SOS, univariate and multivariate analyses were applied. Thereby, *p* values of less than 0.05 indicated statistical significance. The results for each variable were expressed as odds ratios (OR) with their 95% confidence intervals (CI). ROC curve analysis was used to determine adequate cutoffs. Univariate analyses were carried out by chi-square test or Fisher’s exact test. Moreover, the Mann–Whitney *U* test was used to compare the median values of metric variables. Variables that were significant in the univariate analyses were entered into multivariate analysis. The multivariate analysis was performed by backward stepwise logistic regression. All calculations were carried out using the software IBM SPSS Statistics 26.

## Results

### Patient characteristics

The characteristics of the 105 patients are presented in Table 1. The study population consisted of 61 males and 44 females with a median age of 8.6 years (ranged from 0.2 to 26.2 years). Either bone marrow (*n* = 74) or peripheral blood (*n* = 31) was used as the stem cell source. The most frequent underlying diseases were acute lymphoblastic leukemia (*n* = 27), acute myeloid leukemia (*n* = 25) and genetic disease (*n* = 25).

**Table 1:** Characteristics of patients and donors

Characteristics	No
Patients (%)	105 (100)
Median age, years (range)	8.6 (0.2–26.2)
Male (%)	61 (58.1)
Female (%)	44 (41.9)
Donors	
Median age, years (range)	28.5 (0.9–54.9)
Male (%)	63 (60.0)
Female (%)	42 (40.0)
Patients’ diagnoses	
Acute lymphoblastic leukemia (%)	27 (25.7)
Acute myeloid leukemia (%)	25 (23.8)
Myelodysplastic syndrome (%)	14 (13.3)
Lymphoma (%)	2 (1.9)
Solid tumor (%)	12 (11.4)
Genetic disease (%)	25 (23.8)
Stem cell source	
Bone marrow (%)	74 (70.5)
Peripheral blood (%)	31 (29.5)
Type of donors	
HLA-compatible unrelated (%)	52 (49.5)
HLA-mismatched unrelated (%)	20 (19.0)
HLA-haploidentical related (%)	18 (17.1)
HLA-identical related (%)	15 (14.3)

*No.* number; *HLA* human leukocyte antigen

### Incidence and mortality of SOS

SOS occurred in 15 out of 105 transplantations (14.3%). The median time of the SOS diagnosis was 12 days after HSCT (range 1–26 days). Mild SOS occurred in 1 case, moderate SOS in 2 cases, severe SOS in 3 cases while 9 patients showed a very severe form. Among the 15 patients with SOS, 3 subsequently died (20.0%). These 3 patients died within the first 100 days after HSCT. In contrast, 5 out of 90 patients without the diagnosis of SOS died in this period, which results in 100-day mortality of only 5.6%.

### Analysis of risk factors

**Fig. 1:**
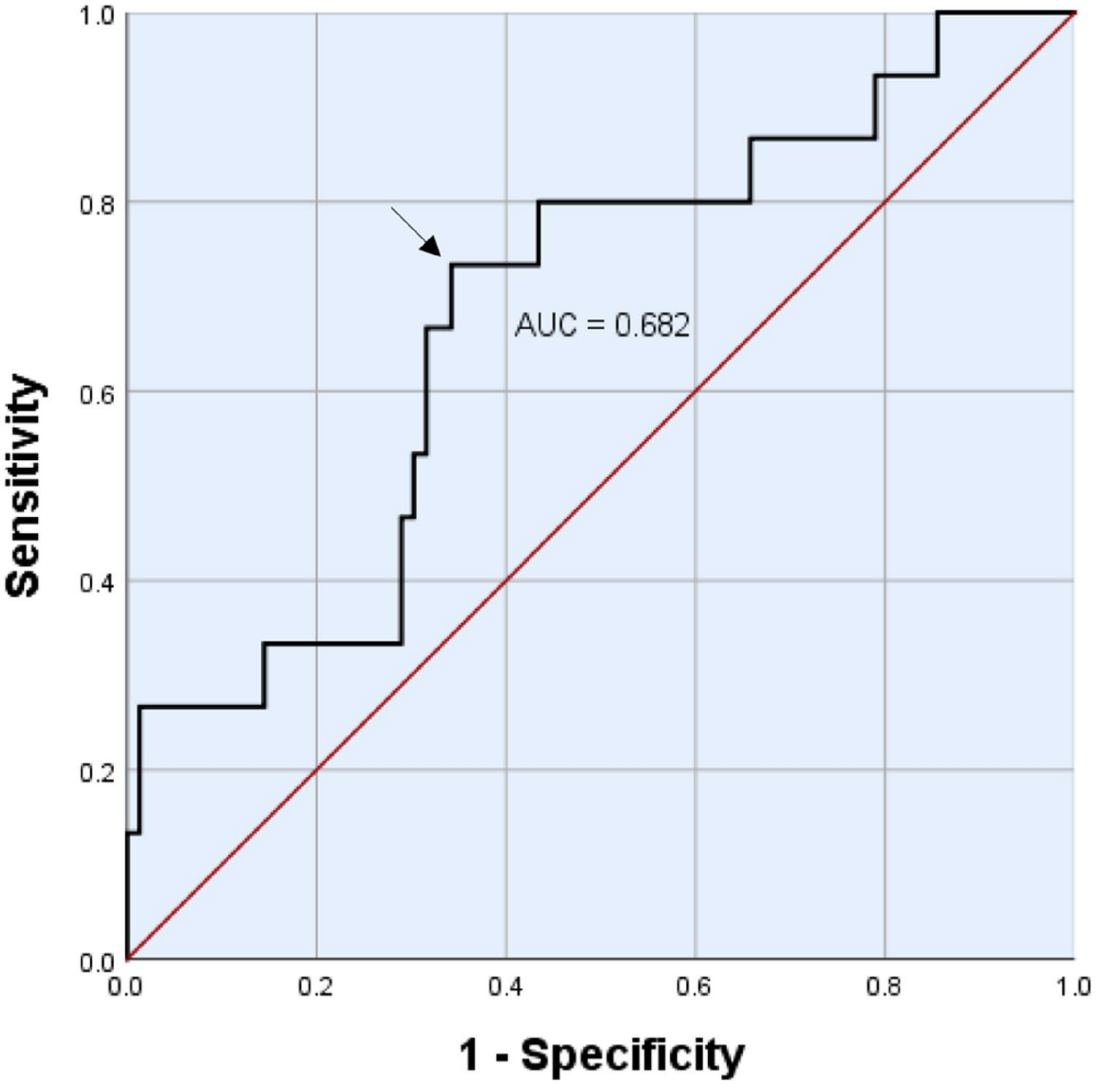
Receiver operating characteristic curve of different ferritin cutoffs. Best ferritin cutoff is marked with an arrow (2420.15 ng/mL); Area under the curve (AUC)

Tables 2 and 3 display the univariate analyses of transplantation-related and patient-related factors. We could not find any significant association between transplantation-related factors and the occurrence of SOS. However, several significant patient-related risk factors could be identified in our study. Patients aged less than 1 year had a significantly higher rate of SOS compared to older patients (50.0% vs. 12.1%, OR = 7.25, *p* = 0.037). Additionally, prior treatment with gemtuzumab ozogamicin was significantly associated with the incidence of SOS (OR = 11.00, *p* = 0.020). The SOS rate in patients treated with gemtuzumab ozogamicin was 60.0% compared to 12.0% in the group without such treatment. By comparing the pretransplant serum levels of ferritin in patients with SOS versus those without SOS, a significantly higher median ferritin was found in patients who developed SOS (2816.9 ng/mL vs. 1554.0 ng/mL, *p* = 0.026). Different cutoffs for serum ferritin were analyzed by a ROC curve (Fig. 1). A cutoff value of 2420.15 ng/mL (see arrow in Fig. 1) indicated the best result for sensitivity (73.3%) and specificity (65.8%). To put this cutoff into clinical practice, it was rounded to a value of 2400 ng/mL. Patients with serum ferritin > 2400 ng/mL showed a significantly higher incidence of SOS compared to those with ferritin ≤ 2400 ng/mL (29.7% vs. 7.4%, OR = 5.29, *p* = 0.005). Furthermore, ferritin > 1500 ng/mL (OR = 4.00, *p* = 0.033) and ferritin > 2000 ng/mL (OR = 4.69, *p* = 0.016) were significant risk factors. Additionally, we noted a significant correlation between pretransplant INR ≥ 1.3 and the occurrence of SOS (OR = 5.91, *p* = 0.009). Patients with INR ≥ 1.3 showed a SOS rate of 37.5%. In contrast, the SOS rate was 9.2% in patients with lower INR.

**Table 2:** Univariate analyses of transplantation-related factors

Transplantation-related factors	No	SOS	OR	95% CI	*p*
Busulfan					
Yes	45	8 (17.8%)	1.64	0.55–4.91	0.376
No	60	7 (11.7%)	–	–	–
Busulfan plus cyclophosphamide or melphalan					
Yes	31	5 (16.1%)	1.23	0.38–3.95	0.764
No	74	10 (13.5%)	–	–	–
Total body irradiation					
Yes	19	4 (21.1%)	1.82	0.51–6.48	0.466
No	86	11 (12.8%)	–	–	–
Stem cell source					
Bone marrow	74	13 (17.6%)	3.09	0.65–14.60	0.221
Peripheral blood	31	2 (6.5%)	–	–	–
Donor age					
≤ 28 years	46	9 (19.6%)	3.24	0.82–12.91	0.082
> 28 years	43	3 (7.0%)	–	–	–
Donor sex					
Female	42	7 (16.7%)	1.38	0.46–4.13	0.569
Male	63	8 (12.7%)	–	–	–
HLA-mismatch					
No	67	10 (14.9%)	1.16	0.36–3.68	0.804
Yes	38	5 (13.2%)	–	–	–

*No.* number; *SOS* sinusoidal obstruction syndrome; *OR* odds ratio; *CI* confidence interval; *HLA* human leukocyte antigen

**Table 3:** Univariate analyses of patient-related factors

Patient-related factors	No	SOS	OR	95% CI	*p*
Patient age					
< 1 year	6	3 (50.0%)	7.25	1.31–40.10	**0.037**
≥ 1 year	99	12 (12.1%)	–	–	–
Patient sex					
Female	44	7 (15.9%)	1.25	0.42–3.76	0.686
Male	61	8 (13.1%)	–	–	–
Gemtuzumab ozogamicin					
Yes	5	3 (60.0%)	11.00	1.67–72.68	**0.020**
No	100	12 (12.0%)	–	–	–
Aspartate transaminase					
> 1 µmol/L*s	10	2 (20.0%)	1.48	0.28–7.83	0.643
≤ 1 µmol/L*s	83	12 (14.5%)	–	–	–
Alanine transaminase					
> 1 µmol/L*s	28	5 (17.9%)	1.46	0.78–10.89	0.538
≤ 1 µmol/L*s	77	10 (13.0%)	–	–	–
Cholinesterase					
< 90 µmol/L*s	21	5 (23.8%)	1.80	0.51–6.30	0.497
≥ 90 µmol/L*s	54	8 (14.8%)	–	–	–
Glutamyl transpeptidase					
≤ 0.5 µmol/L*s	44	9 (20.5%)	2.11	0.65–6.88	0.210
> 0.5 µmol/L*s	46	5 (10.9%)	–	–	–
Lactate dehydrogenase					
> 5 µmol/L*s	9	2 (22.2%)	1.78	0.33–9.52	0.616
≤ 5 µmol/L*s	94	13 (13.8%)	–	–	–
Alkaline phosphatase					
> 3 µmol/L*s	13	4 (30.8%)	3.38	0.88–13.03	0.085
≤ 3 µmol/L*s	86	10 (11.6%)	–	–	–
Ferritin					
> 2400 ng/mL	37	11 (29.7%)	5.29	1.53–18.25	**0.005**
≤ 2400 ng/mL	54	4 (7.4%)	–	–	–
Ferritin					
> 2000 ng/mL	47	12 (25.5%)	4.69	1.22–17.95	**0.016**
≤ 2000 ng/mL	44	3 (6.8%)	–	–	–
Ferritin					
> 1500 ng/mL	50	12 (24.0%)	4.00	1.05–15.32	**0.033**
≤ 1500 ng/mL	41	3 (7.3%)	–	–	–
Albumin					
< 30 g/L	62	9 (14.5%)	1.02	0.33–3.11	0.974
≥ 30 g/L	42	6 (14.3%)	–	–	–
Total bilirubin					
> 17 µmol/L	28	5 (17.9%)	1.46	0.45–4.71	0.538
≤ 17 µmol/L	77	10 (13.0%)	–	–	–
C-reactive protein					
> 18 mg/L	40	8 (20.0%)	2.07	0.69–6.24	0.189
≤ 18 mg/L	65	7 (10.8%)	–	–	–
International normalized ratio					
≥ 1.3	16	6 (37.5%)	5.91	1.65–21.19	**0.009**
< 1.3	76	7 (9.2%)	–	–	–

*p*-values of less than 0.05 indicated statistical significance

*No.* number; *SOS* sinusoidal obstruction syndrome; *OR* odds ratio; *CI* confidence interval

As presented in Table 4, the following factors were significant in our multivariate analysis: prior treatment with gemtuzumab ozogamicin (OR = 9.24, *p* = 0.048), ferritin > 2400 ng/mL (OR = 5.74, *p* = 0.023) and INR ≥ 1.3 (OR = 8.02, *p* = 0.007).

**Table 4:** Multivariate analysis of risk factors

Risk factors in multivariate analysis	OR	95% CI	*p*
Gemtuzumab ozogamicin	9.24	1.02–83.55	**0.048**
Ferritin > 2400 ng/mL	5.74	1.27–26.04	**0.023**
INR ≥ 1.3	8.02	1.77–36.43	**0.007**

*p*-values of less than 0.05 indicated statistical significance

*OR* odds ratio; *CI* confidence interval; *INR* International normalized ratio

## Discussion

In our study, 15 out of 105 patients developed SOS. Consequently, the incidence of SOS was 14.3%. In a previous study, which compared different incidence rates of SOS across several studies an overall mean incidence of 13.7% was reported (Coppell et al. 2010). This demonstrates that our result is consistent with previous data. However, Kammersgaard et al. (2019) showed a higher SOS incidence of 44.8% in a population of 87 children. In that study, pediatric EBMT criteria were used corresponding to our study. Further studies with a larger study population are necessary to specify the incidence of SOS defined by the pediatric EBMT criteria. In our study population, the median time of SOS onset was 12 days after HSCT, which corresponds to the literature. Yakushijin et al. (2016) retrospectively analyzed 4290 patients who underwent allogeneic HSCT. In that study, the median time of SOS diagnosis was also 12 days post-HSCT (Yakushijin et al. 2016). From all patients with SOS, we observed a relative distribution of 6.7% mild, 13.3% moderate, 20.0% severe, and 60.0% very severe disease courses. In the current literature, similar results can be found when the EBMT grading criteria were used. Yoon et al. (2019) reported a rate of 5.9% mild, 12.8% moderate, 18.2% severe, and 63.1% very severe courses of SOS. In a study published by Kammersgaard et al. (2019), 7.7% had mild SOS, 15.4% had moderate SOS, 43.6% had severe SOS, and 33.3% showed very severe SOS.

In the present study, the mortality rate from SOS was 20.0%, which is a lower rate, especially compared to older studies (Barker et al. 2003; Jones et al. 1987; McDonald et al. 1993). The lower mortality rates in recent studies are probably due to the early therapy with defibrotide (Corbacioglu et al. 2016; Mohty et al. 2020).

Previous publications have already shown significant associations between the transplantation-related factors of conditioning regimen based on busulfan or total body irradiation and the occurrence of SOS (Barker et al. 2003; Cheuk et al. 2007; Yakushijin et al. 2016). On the contrary, these reported risk factors were not found to be significant in our study. One reason for this can be the limited number of analyzed patients. Nevertheless, also other studies could not find a significant correlation (Kami et al. 1997; Maximova et al. 2014).

A significant relationship between an increased risk of SOS and donor mismatch has already been reported (Hasegawa et al. 1998). We could not confirm this finding in our patient population. Other transplantation-related factors like stem cell source, donor age, and donor sex were not significantly associated with the incidence of SOS either. Carreras et al. (2011) showed a significantly higher rate of SOS in transplantations with bone marrow stem cells compared to transplantations with peripheral blood stem cells. A few other analyses could not find a significant correlation between the stem cell source and the development of SOS (Soyer et al. 2020; Strouse et al. 2018). In future trials, this potential risk factor should be further explored.

With regards to younger patients, we found out that an age < 1 year had a significant impact on the development of SOS in the univariate analysis (*p* = 0.037). Full hepatic maturity takes up to 2 years after birth (Beath 2003). This demonstrates that infants have a reduced hepatic detoxification function, which consequently makes them particularly vulnerable to conditioning regimens. Thus, higher rates of SOS could be explained. Moreover, pediatric diseases that are predisposing to SOS are found more often in the first years of life (Cesaro et al. 2005). This especially applies to neuroblastoma. Our findings concur with the published literature although different cutoffs for age were proposed (Cesaro et al. 2005; Cheuk et al. 2007; Faraci et al. 2019).

In our study, we could not find a significant correlation between female sex and the incidence of SOS. According to our results, this factor was not listed in some detailed reviews (Cairo et al. 2020; Dalle and Giralt 2016). However, other studies identified female sex as a significant risk factor (Faraci et al. 2019; Hägglund et al. 1998). This aspect should be further investigated in future trials.

Previous reports have already highlighted the treatment with gemtuzumab ozogamicin as a risk factor for SOS incidence (Richardson and Corbacioglu 2020; Wadleigh et al. 2003). It is assumed that gemtuzumab ozogamicin targets CD33 + cells in the hepatic sinusoids, such as Kupffer cells, stellate cells, and endothelial cells (Rajvanshi et al. 2002). Our study confirms the significant risk factor of prior treatment with gemtuzumab ozogamicin for the pediatric population in univariate analysis (*p* = 0.020) as well as in multivariate analysis (*p* = 0.048).

In the literature, some studies showed significant correlations between SOS and elevated values of aspartate transaminase, alanine transaminase, and total bilirubin as well as reduced values of cholinesterase and albumin (Carreras et al. 1998; Hägglund et al. 1998; Hasegawa et al. 1998; Srivastava et al. 2004). These values indicate preexisting liver damage. However, we could not find such significant associations in our patient population. According to the current state of relevant studies, the laboratory parameters of glutamyl transpeptidase, lactate dehydrogenase, alkaline phosphatase, and C-reactive protein were not significant risk factors (Dalle and Giralt 2016). In regard to serum ferritin, we detected significantly higher SOS rates in patients with ferritin > 1500 ng/mL, > 2000 ng/mL and > 2400 ng/mL. However, > 2400 ng/mL was the optimal cutoff with *p* = 0.005 in univariate analysis and *p* = 0.023 in multivariate analysis. High serum ferritin indicates iron overload, which is considered to be a reason for liver dysfunction (McKay et al. 1996; Miceli et al. 2006). Iron induces the development of oxygen free radicals that lead to an injury of hepatic tissue (Ramm and Ruddell 2005). Additionally, high serum ferritin can be explained by the response to inflammation through its role as an acute-phase protein (Armand et al. 2012). It can be suggested that these factors predispose to SOS. Our findings accord with other studies (Maradei et al. 2009; Maximova et al. 2014; Morado et al. 1999).

In our study, we report for the first time that high pretransplant INR was significantly associated with the occurrence of SOS. The cutoff of ≥ 1.3 was significant in univariate analysis (*p* = 0.009) as well as in multivariate analysis (*p* = 0.007). Higher INR values indicate increased bleeding tendency (Kirkwood 1983). Although SOS is characterized by downstream embolization and sinusoidal obstruction, there is an initial hemorrhage of erythrocytes, leukocytes, and cellular debris into the spaces of Disse (Carreras and Diaz-Ricart 2011; Mohty et al. 2015). This is why increased bleeding tendency, measured by high INR, could lead to a higher risk of SOS. Moreover, INR is affected by vitamin K-dependent coagulation factors (Tripodi et al. 1995). High INR can be caused by a lack of coagulation factors, which is linked with liver dysfunction. This is another reason why high INR might be correlated to SOS.

Our study is limited by the relatively small sample size, which leads to reduced statistical power. In some cases, data were missing. Because of this, not all 105 patients could be included in each analysis. However, the rather small number of patients is not uncommon in single-center studies with only pediatric patients. Additionally, our study is a retrospective analysis. Therefore, it is more susceptible to observation and selection bias compared to prospective studies. Nonetheless, inclusion criteria were clearly defined and consistently applied.

The diagnosis of SOS was based on the pediatric criteria according to Corbacioglu et al. (2018) because they are strongly recommended by the EBMT. Patients who had been transplanted before the pediatric EBMT criteria were published needed to be re-evaluated. Thus, consistent criteria were applied. Future analyses should standardly use the EBMT criteria to avoid retrospective re-evaluation.

In conclusion, our findings confirm the risk factors of young patient age (< 1 year), prior treatment with gemtuzumab ozogamicin, and high serum ferritin (> 2400 ng/mL) for children and AYA. Furthermore, the significant association between high pretransplant INR (≥ 1.3) and the development of SOS is reported for the first time. Our findings can contribute to better risk stratification and a modified screening system after allogeneic HSCT in pediatric patients. Finally, further studies are necessary to validate our findings. This especially applies to the new risk factor of high INR.
